# The Spatial Extent of Pain Is Associated with Pain Intensity, Catastrophizing and Some Measures of Central Sensitization in People with Frozen Shoulder

**DOI:** 10.3390/jcm11010154

**Published:** 2021-12-28

**Authors:** Mercè Balasch-Bernat, Lirios Dueñas, Marta Aguilar-Rodríguez, Deborah Falla, Alessandro Schneebeli, Marta Navarro-Bosch, Enrique Lluch, Marco Barbero

**Affiliations:** 1 Physiotherapy in Motion, Multi-Speciality Research Group (PTinMOTION), Department of Physiotherapy, University of Valencia, 46010 Valencia, Spain; merce.balasch@uv.es (M.B.-B.); marta.aguilar@uv.es (M.A.-R.); enrique.lluch@uv.es (E.L.); 2 Centre of Precision Rehabilitation for Spinal Pain (CPR Spine), School of Sport, Exercise and Rehabilitation Sciences, College of Life and Environmental Sciences, University of Birmingham, Edgbaston, Birmingham B15 2TT, UK; d.falla@bham.ac.uk (D.F.); alessandro.schneebeli@supsi.ch (A.S.); 3 Rehabilitation Research Laboratory, Department of Business Economics, Health and Social Care, University of Applied Sciences and Arts of Southern Switzerland, SUPSI, Stabile Piazzetta, Via Violino, 6928 Manno, Switzerland; marco.barbero@supsi.ch; 4 Servicio de Cirugía Ortopédica y Traumatología del Hospital Universitari I Politècnic La Fe de València, 46026 Valencia, Spain; mnavarrob26@yahoo.es; 5 Pain in Motion Research Group (PAIN), Department of Physiotherapy, Human Physiology and Anatomy (KIMA), Faculty of Physical Education & Physiotherapy, Vrije Universiteit Brussel, B-1050 Brussels, Belgium

**Keywords:** pain drawings, pain extent, pain location, frozen shoulder, pain sensitization, psychological factors

## Abstract

The aim of this cross-sectional study was to explore the spatial extent of pain and its association with clinical symptoms, psychological features, and pain sensitization in people with frozen shoulder (FS). Forty-eight individuals with FS completed pain drawings (PDs) and reported their clinical symptoms including pain intensity (Visual Analogue Scale) and shoulder disability (Shoulder Pain and Disability Index). Moreover, pain sensitization measurements (pressure pain thresholds, temporal summation, conditioned pain modulation, and Central Sensitization Inventory (CSI)) were assessed. Psychological features were assessed by Pain Catastrophizing Scale (PCS) and Pain Vigilance and Awareness Questionnaire. Pain frequency maps were generated, Margolis rating scale was used for pain location, and Spearman correlation coefficients were computed. The mean (SD) pain extent was 12.5% (6.7%) and the most common painful area was the anterolateral shoulder region (100%). Women presented a more widespread pain distribution compared with men. Significant positive associations were obtained between pain extent and current pain intensity (rs = 0.421, *p* < 0.01), PCS (rs = 0.307, *p* < 0.05) and CSI (rs = 0.358, *p* < 0.05). The anterolateral region of the shoulder was the most common painful area in people with FS. Women with FS presented more extended areas of pain; and a more widespread distribution of pain was correlated with higher levels of pain, pain catastrophizing and pain sensitization.

## 1. Introduction

Frozen shoulder (FS) is a frequent musculoskeletal disorder which is characterized by progressive loss of shoulder range of motion [1]. The prevalence of FS in the general population is 2% to 5% and women aged between 40 and 65 years are more commonly affected [2]. FS has been classified into primary, characterized by an insidious onset and an idiopathic origin, and secondary, which seems to be associated with a certain event, either a systemic (i.e., diabetes), intrinsic (i.e., rotator cuff disease) or extrinsic (i.e., cervical radiculopathy) cause [3,4]. In addition to pain and movement restriction, FS often leads to sleep deprivation, anxiety, high levels of disability and has an important impact on nearly every aspect of daily living [5].

To a large extent, FS has been traditionally considered a clinical condition where the dominant operating pain mechanism is assumed to be nociceptive [6]. Nevertheless, recent research suggests that this condition has a complex pathophysiology, which has not yet been fully understood. On the one hand, some authors have argued that central pain mechanisms might have a relevant role in a subgroup of patients with FS [7]. Taking into account that FS is characterized by being long-lasting, it is plausible that neuroplastic changes occurring at different levels of the central nervous system might contribute to pain maintenance [8]. Apart from some preliminary research [9,10,11,12,13], the implication of central pain mechanisms in people with FS remains uncertain. On the other hand, the role of low-grade inflammation and immune system response dysregulation leading to pathological changes in the shoulder capsule is gaining interest in the scientific literature [2,3]. In this sense, a state of low-grade inflammation seems to be a contributing factor to the development of FS. In particular, at early stages of the pathology, an immune response with elevated levels of alarmins has been found in the shoulder capsule of people with FS, triggering a cascade of inflammation and leading to subsequent fibrosis of the capsular tissue [14]. These mechanisms appear to be perpetuated by an upregulated production of pro-inflammatory cytokines and a neuro-immune activation [15,16]. Additionally, low-grade inflammation is one of the factors that may be involved in the development of an increase in excitability of the central nervous system (i.e., central sensitization) [17,18]. A deeper understanding of pain mechanisms is considered essential for reaching accurate diagnosis and treatment approaches [19].

The contribution of central pain mechanisms in patients suffering from chronic musculoskeletal pain has been inferred by the presence of widespread pain as identified from a body chart [20]. Indeed, a widespread pain distribution is a key sign of altered central pain processing mechanisms [21,22]. In line with this, pain drawings (PDs) have been used to assess clinical features of central sensitization [23,24] and to quantify pain extent and pain location in a wide variety of musculoskeletal pain conditions such as neck pain [25], whiplash [26], migraine [27], low back pain [28], fibromyalgia [29], and knee [22,30] and hip osteoarthritis [31], among others. However, to the authors’ knowledge, PDs have not yet been used in people with FS. Firstly, the implementation of PDs in people with FS may help to establish the typical pain distribution in this population. Secondly, PDs can be considered as an easy and cheap method for the identification of altered central pain mechanisms in patients with FS. Thus, PDs may indirectly provide clinicians with information regarding pain mechanisms and guide them to an early identification of FS patients presenting with central sensitization without the need for expensive equipment.

Furthermore, it is well known that psychological and social factors contribute to symptoms and disability in chronic musculoskeletal pain [32,33]. Some studies have demonstrated that chronic pain is associated with psychological aspects such as fear-avoidance beliefs [34], pain catastrophizing [35], or emotional distress [36]. Moreover, associations between particular personality traits and FS have recently been found by Chiaramonte et al. [37], indicating an interaction between psychological and somatic factors. Although there is extensive use of PDs in clinical practice, research regarding how pain location and extent are associated with psychological factors in patients with FS is lacking.

Therefore, the primary aim of the current study was to quantify pain location and spatial extent of pain in subjects with FS by means of a digital PD. As secondary aims, the association between the extent of pain and clinical symptoms, psychological features and pain sensitization were investigated.

## 2. Materials and Methods

### 2.1. Study Design

This is a cross-sectional observational study, which was conducted at the pain research unit of the University of Valencia (Valencia, Spain) between October 2015 and December 2017. This study is reported in line with the Strengthening the Reporting of Observational Studies in Epidemiology (STROBE) statement [38]. It was approved by the Human Research Ethics Committee of the University of Valencia (protocol number H1432625002427) and complies with the Declaration of Helsinki.

### 2.2. Participants

A sample of 48 participants with primary FS, diagnosed by a physician, were recruited from different outpatient private clinics and rehabilitation services of several hospitals of the region of Valencia, and from community-based advertisements. Before the commencement of the study, an informed consent was obtained from all participants.

Primary FS was diagnosed on the basis of the following inclusion criteria: (1) a reduction in passive external rotation greater than 50% compared with the unaffected shoulder or an external rotation lower than 30° [39]; (2) loss of range of motion greater than 25% in at least two planes of movement when compared with the unaffected shoulder [39]; (3) pain and movement restriction had to be present for at least 1 month, having reached a plateau or worsened [39]; and (4) normal shoulder X-rays (except for osteopenia of the humeral head and calcific tendinosis) [4]. In addition, to be included in this study, participants had to be stable in medication intake for at least 4 weeks prior to the study commencement [40]. People with secondary FS, locked dislocations, arthritis, fractures, avascular necrosis, cervical radiculopathy or previous surgery in the upper quadrant region during the last year, were excluded. Participants were also excluded if they had conditions characterized by dominant central pain mechanisms (i.e., fibromyalgia), rheumatic diseases, co-morbidities (i.e., cardiovascular, cognitive or neurological diseases), or were taking centrally acting analgesics (i.e., antidepressants and anticonvulsants).

### 2.3. Procedure

Participants’ characteristics, including age, sex, weight, height, and pain duration, were collected at baseline. Additionally, participants quantified their pain intensity and were asked to complete a PD to report their painful area. Then, participants completed the following self-administered questionnaires: Central Sensitization Inventory (CSI) [41], Shoulder Pain and Disability Index (SPADI) [39], Pain Catastrophizing Scale (PCS) [42], and Pain Vigilance and Awareness Questionnaire (PVAQ) [43]. Finally, participants were assessed for pain sensitization [44] using Quantitative Sensory Testing (QST). Specifically, pressure pain thresholds (PPTs), temporal summation (TS), and conditioned pain modulation (CPM) were measured.

All assessments were performed in a single session and always in the same order. Participants were asked not to take any analgesic medication 24 h before the examination. The researcher performing QST ensured that all participants met this requirement by asking them about medication intake prior to the measurements. This researcher was blinded to the questionnaires and PDs data.

### 2.4. Pain Extent

The spatial extent of pain was quantified by using a method for PD acquisition which had previously been shown to be reliable in people with chronic neck and low back pain [45]. PDs were performed on a digital tablet (iPad 2, Apple Inc., Cupertino, CA, USA) with a stylus pen for digital tablets (CS100B, Wacom Technology Corp, Vancouver, Washington) and a commercially available sketching software (SketchBook Pro, Autodesk Inc., San Rafael, CA, USA). In accordance with the participant’s sex, a male or female body chart was chosen with different views of the shoulder region (i.e., frontal and dorsal) and opened in the sketching software. The type, size, and color of the pen stroke were standardized across all participants.

An examiner, who was trained in the use of the PD acquisition software prior to the study commencement, gave each participant a standardized verbal explanation about how to complete the PD with the digital tablet. The PD was presented to participants as a tool where they should accurately illustrate where they had felt pain during the previous week. The assessor highlighted the importance of fully representing all pain locations. After a short demonstration to familiarize the participants with the device, they were required to complete their PDs. Participants were instructed as follows: “Please shade the area/s where you felt your usual pain during the last week on this body chart and try to be as precise as possible.” They were instructed to color every part of the body where they felt pain in the previous week, regardless of the quality and the severity of pain. Before saving and storing the PD, participants were asked whether the colored PD corresponded to their real pain distribution. If not, they were given the possibility to correct the PD using the “eraser” tool.

A custom-designed software program was used to calculate the total pain extent for each participant and to generate two pain frequency maps (i.e., frontal and dorsal body chart) separately for men and women [45]. Pain extent and pain location were scored using the Margolis rating scale [46]. For each anatomical region affected by pain, a pain percentage was attributed. Thus, scores from the dorsal view and frontal view were combined to generate a single value of pain extent (i.e., total pain extent).

### 2.5. Pain Sensitization

#### 2.5.1. Pressure Pain Thresholds (PPTs)

A standardized protocol for evaluating PPTs was used [47]. PPTs were measured at the middle deltoid of the affected shoulder, 2 cm below the lateral part of the acromion, using an analog hand-held pressure algometer (Wagner Instruments, FDIX; Wagner Instruments, Greenwich, CT, USA) with a surface area at the round tip of 1 cm^2^. The algometer probe tip was applied perpendicular to the skin at a rate of 1 kg/cm^2^/s until the first onset of pain. PPTs were measured three times, with a 30 s interstimulus interval between measurements, and the mean was used for statistical analysis. PPTs are a reliable measure of deep tissue sensitivity [48] and have been extensively investigated in people with shoulder pain [40,49].

#### 2.5.2. Temporal Summation (TS) and Conditioned Pain Modulation (CPM)

For measuring excitability of nociceptive pathways and efficacy of endogenous pain inhibition, the TS and CPM paradigms were used [50,51].

First, PPTs were measured at the middle deltoid of the affected shoulder, as described above. After 2 min rest, TS was provoked by means of 10 consecutive pulses at the previously determined PPT. For each pulse, pressure was gradually increased at a rate of 2 kg/s to the determined PPT and maintained for 1 s before being released (1 s interstimulus interval). Pain intensity of the 1st, 5th, and 10th pulses were rated by participants on a numerical rating pain scale (0 = “no pain” to 10 = “worst possible pain”).

After a rest period of 5 min, CPM was induced by combining the PPTs procedure (namely, the test stimulus) with an inflated occlusion cuff around the participant’s arm, in the unaffected shoulder (conditioning stimulus). The occlusion cuff was inflated at a rate of 20 mm Hg/s until “the first sensation of pain” and maintained for 30 s. Then, intensity of pain, as a result of cuff inflation, was rated on a numerical rating pain scale. Afterward, cuff inflation was increased or decreased until the intensity of pain was rated as 3/10. The length of time to reach 3/10 pain was recorded. PPTs assessment was then repeated during maintenance of the cuff inflation. Details and data supporting test–retest reliability and validity of the protocols for examining TS and CPM can be found elsewhere [52,53].

#### 2.5.3. Central Sensitization Inventory (CSI)

The CSI is a questionnaire useful to quantify the severity of several symptoms of central sensitization, which has been shown to be valid and reliable [54]. Part A of the CSI includes 25 items related to symptoms common to central sensitization, each rated on a 5 point scale with the end points 0 (“never”) and 4 (“always”) (range 0–100). The following CSI severity levels have been established: subclinical = 0 to 29; mild = 30 to 39; moderate = 40 to 49; severe = 50 to 59; and extreme = 60 to 100 [55]. The Spanish version of the CSI was used in this study [41].

### 2.6. Clinical Symptoms

#### 2.6.1. Pain Intensity

Current shoulder pain intensity in addition to average shoulder pain over the last 24 h were recorded using a 100 mm Visual Analogue Scale (VAS) ranging from 0 (“no pain”) to 100 (“worst imaginable pain”). The VAS is a reliable and valid tool commonly used to assess pain intensity [56]. The minimal clinically important difference (MCID) for the VAS is estimated to be 30 mm [57].

#### 2.6.2. Shoulder Pain and Disability

Shoulder pain related disability was measured with the Spanish version of the Shoulder Pain and Disability Index (SPADI) [58]. The SPADI consists of 13 items distributed in two domains: pain (5 items, range 0–50 points) and disability (8 items, range 0–80 points). All items are scored using a numeric rating scale ranging from 0 (“no pain/no difficulty”) to 10 (“worst pain imaginable/so difficult it required help”). Scores from both pain and disability subscales are averaged to calculate the total score (0–100 points). A higher score indicates greater shoulder pain related disability [59]. Psychometric properties of the Spanish version of the SPADI have been shown to be acceptable for clinical use, with high internal consistency (Cronbach α: 0.916) and excellent test–retest reliability (ICC: 0.91) [58,60]. Additionally, the minimal detectable change (MDC) has been determined to be 18, whereas the minimal clinically important difference (MCID) ranges between 8 and 13 [60].

### 2.7. Psychological Features

#### 2.7.1. Pain Catastrophizing

The Pain Catastrophizing Scale (PCS) is a self-administered questionnaire comprising 13 items structured into 3 dimensions: rumination, magnification, and helplessness [61]. Each item is rated from 0 (“not at all”) to 4 (“all the time”) (range 0–52), with higher scores indicating increased pain catastrophizing. The Spanish version of the PCS used in this study showed good psychometric properties in people with fibromyalgia [42].

#### 2.7.2. Pain Hypervigilance

The Spanish version of the Pain Vigilance and Awareness Questionnaire (PVAQ) was used to assess participants’ preoccupation with, or attention to, pain associated with pain-related fear and perceived severity of pain [43]. The PVAQ comprises 9 items, each rated on a 6 point scale with the end points 0 (“never”) and 5 (“always”) (range 0–45). Higher scores indicate a higher degree of pain vigilance and awareness. Psychometric properties of the PVAQ have been previously reported in people with chronic back pain [43] and fibromyalgia [62], showing appropriate internal consistency, reliability, and validity.

### 2.8. Data Analysis

Data distribution was initially assessed with the Shapiro–Wilk test, and non-normally distributed data were identified. Descriptive statistics were used to present the clinical and demographic characteristics of the participants with FS. Pain frequency maps were generated by superimposing the PDs from all participants to illustrate the most frequently reported location of pain across the entire sample. This was performed for women and men separately. Pain distribution in the anatomical regions was reported using histograms. Spearman correlation coefficients were computed to reveal possible correlations between: (1) the area of pain and pain sensitization (i.e., PPTs, TS, CPM and CSI), (2) the area of pain and clinical symptoms (i.e., pain duration, VAS and SPADI), and (3) the area of pain and psychological features (i.e., PCS and PVAQ). The strength of the correlations was interpreted as follows: no correlation (0.00–0.25), low (0.26–0.49), moderate (0.50–0.69), high (0.70–0.89), and very high (0.90–1.00) [63]. Statistical analysis was performed using IBM SPSS version 24 (IBM Corp, Armonk New York) and the level of significance was set at *p* < 0.05.

## 3. Results

**Figure 1:**
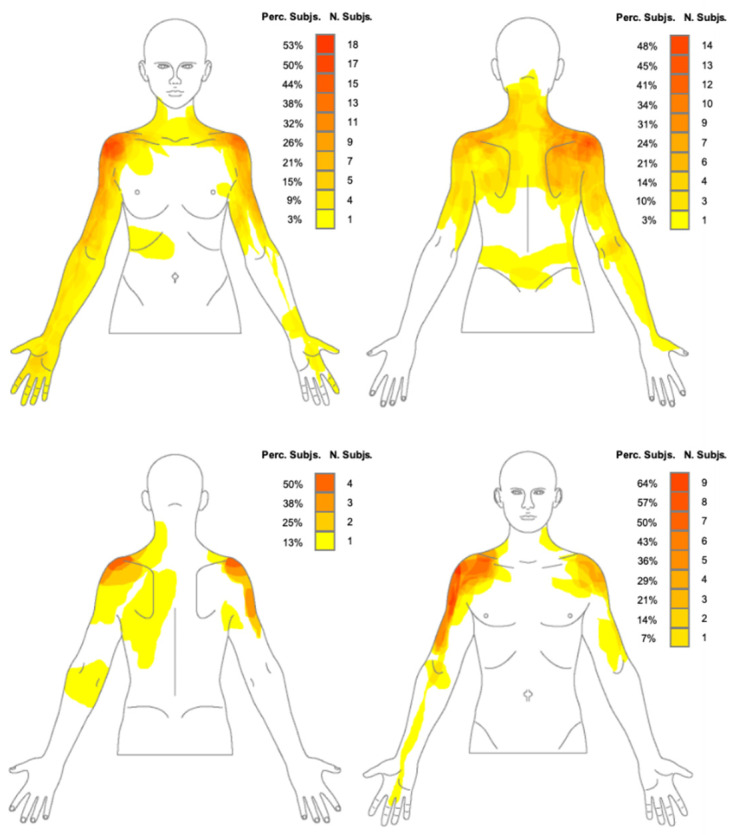
Pain frequency and pain location maps in men and women with frozen shoulder. The color grid indicates both the number and the percentage of participants reporting pain in that specific area. Dark red represents the most frequently reported painful area.

Forty-eight individuals with FS (33 women and 15 men) participated in this study. Participants’ demographic characteristics, clinical symptoms, psychological features and pain sensitization measurements are reported in Table 1. Correlations between pain extent and measures of pain sensitization, clinical symptoms and psychological features are detailed in Table 2. The mean (SD) pain extent of the sample was 12.5% (6.7). Pain frequency and location maps, with frontal and dorsal views, displayed separately for men and women, are illustrated in Figure 1. The most common location of pain in both men and women was the anterolateral region of the shoulder. Women presented a more widespread distribution of pain, including the whole upper limb, compared with men.

**Figure 2:**
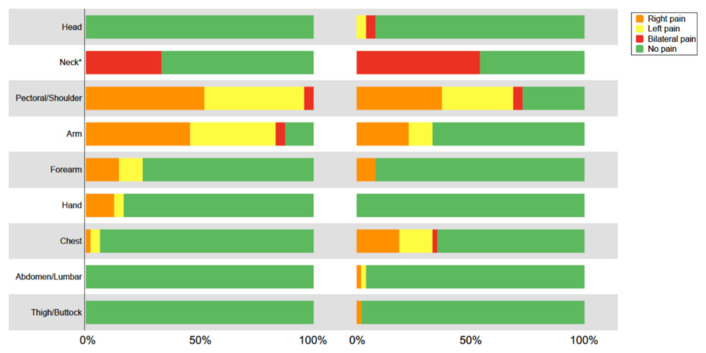
Pain distribution in anatomical regions reported in frontal and dorsal body charts among people with frozen shoulder contracture (*N* = 48). * The neck region was not divided into left and right sides.

According to results from the Margolis rating scale, all participants reported pain in the anterior shoulder region. A significant percentage of patients reported pain in adjacent regions such as the scapular region (72.9%), the anterior portion of the arm (87.5%) and the posterior neck region (54.2%) (Figure 2).

### 3.1. Correlations between Pain Extent and Pain Sensitization

No statistically significant correlations were observed between pain extent and PPTs at the affected shoulder (*r_s_* = −0.118), TS (*r_s_* = −0.149) or CPM (*r_s_* = −0.138), while a low positive correlation was found between pain extent and CSI (*r_s_* = 0.358, *p* < 0.05); participants with a larger pain extent showed higher scores in the CSI.

### 3.2. Correlations between Pain Extent, Clinical Symptoms and Psychological Features

No significant correlation was found between pain extent and pain duration (*r_s_* = 0.195). Pain extent demonstrated a low positive correlation with current pain intensity (*r_s_* = 0.421, *p* < 0.001) and PCS (*r_s_* = 0.307, *p* < 0.05); participants with higher levels of pain and higher pain catastrophizing showed a larger pain distribution. No significant correlations were found between pain extent and shoulder disability (SPADI) (*r_s_* = 0.182), PVAQ (*r_s_* = −0.252) or pain during the last 24 h (*r_s_* = 0.057).

## 4. Discussion

In this study, the association between pain extent and different clinical and pain variables was explored for the first time in people with FS. The results indicate that the most frequent site of pain in people with FS was the anterolateral region of the shoulder. Women with FS reported more extended areas of pain compared with men. Additionally, enlarged areas of pain were positively associated with higher levels of pain intensity, pain catastrophizing and pain sensitization measured with the CSI.

The most common site of symptoms reported by the sample was the anterolateral region of the shoulder. Previous studies have explored the pain distribution in other shoulder pain populations. For instance, Gumina et al. [64] investigated the area of pain reported by patients with rotator cuff tears and found that most patients (86%) felt their pain in the anterolateral aspect of the shoulder with radiation down the lateral surface of the arm to the elbow, while the pain extended to the forearm in only 13% of patients. Bayam et al. [65] investigated pain distribution in people with different shoulder diagnoses, but did not include patients with FS. The authors found that in patients with impingement syndrome, rotator cuff tear and glenohumeral joint osteoarthritis, pain radiated from the shoulder to the forearm, whereas in patients with shoulder instability, acromioclavicular joint pathology and calcific tendonitis, pain was more localized around the shoulder and upper arm. If the current study’s results are compared with the above-mentioned studies, then the distribution of pain described by people with FS is not specific for this population, but involves similar anatomical regions to other shoulder conditions. This suggests that PDs by themselves are likely not enough to establish a pathoanatomical diagnosis in a patient with shoulder pain. Future studies may compare the pain distribution of different shoulder pain populations to determine whether PDs can be helpful in the diagnosis of shoulder pain.

Interestingly, in addition to local shoulder symptoms, many patients with FS also reported enlarged and remote areas of pain, as shown in Figure 1. This spreading of pain to larger areas may reflect an increase in pain sensitization in these individuals, as previous literature suggests that extension of pain is a phenomenon attributed to central sensitization [21,22]. Enlarged areas of pain were noticed in women compared with men in the present study, including the whole upper limb. The results of this current study contrast with previous studies performed in patients with shoulder pain where no sex differences in terms of pain distribution were found [64,65]. However, other studies conducted in other musculoskeletal chronic pain populations [30,31] also found women reporting larger pain areas than men. This could respond to a greater degree of pain sensitization in women. In line with this, previous literature has suggested that women have an increased pain sensitivity to standard stimuli [30,66,67].

Enlarged areas of pain in people with FS were positively associated with higher levels of pain intensity but not with pain duration or shoulder disability. Previous studies focused on PDs also found positive associations between pain extent and pain intensity [30,45]. Although the area of pain and pain intensity are outcomes assessing different constructs, it could be expected that people with FS with a more extended area of pain would report higher pain intensity. Contrary to this study’s results, more widespread areas of pain were found in individuals with knee osteoarthritis pain, particularly in those with more persistent symptoms [68]. Other authors [29] however have found a negative correlation between pain extent and pain duration, with larger pain distributions associated with a shorter history of symptoms. In contrast to this study’s findings, some studies have previously observed significant correlations between pain extent and disability in people with chronic neck pain [45,69]. These inconsistencies in research highlight the importance of being cautious when drawing firm conclusions about patients’ pain duration, pain intensity or disability levels only based on a PD.

The results of this study showed no significant association between pain extent and pain sensitization outcomes (PPTs, TS and CPM) with the exception of the CSI. Although a positive association between pain extent and pain sensitization was expected, this was not the case for most of the outcome, which was in accordance with results obtained by Lluch et al. [30] in people with knee osteoarthritis. Similar to this current study, these authors also found that larger pain areas were positively associated with higher scores in the CSI [30]. PDs as a variable measuring pain distribution and pain sensitization related outcomes (i.e., QST, CSI) assess different constructs related to patients’ pain experience. This may justify why the areas of pain, as assessed with PDs, were not correlated with different biomarkers of pain sensitization such as PPTs, CPM or TS in the present study. In contrast with this study’s findings, negative correlations between pain extent and PPTs were demonstrated in people with knee [30] and hip [31] osteoarthritis. However, Barbero et al. [29] found no associations between pain extent and PPTs in people with fibromyalgia. Similarly, contrasting results were found in the literature about the relationship between pain extent and CPM or TS. Whereas no significant associations were reported between the area of pain and CPM or TS [30], other authors found higher pain extent to be associated with lower CPM in people with non-specific chronic back pain and fibromyalgia [70] and spinal cord injury patients [71]. Further research performed in larger pain population samples is needed to unravel the association, if any, between PDs and pain sensitization outcomes.

Regarding psychological features, only pain catastrophizing was positively correlated with pain extent in the current study, which was in accordance with results obtained by Willett et al. [31] in patients with hip osteoarthritis. Contrary to the current study’s results, previous research in non-shoulder pain populations demonstrated no correlation between the area of pain and the individual psychological state [30,72]. Indeed, a systematic review by Carnes et al. [73] did not support the assumption that unusual or extensive PDs may indicate a disturbed psychological state and concluded that the use of PDs is not recommended for assessing the psychological status of a patient. Other studies found significant correlations between pain extent and other psychological features such as anxiety and depression [45,74] or self-efficacy [45]. A recent systematic review exploring the association between PDs and psychological factors in patients suffering from chronic musculoskeletal pain was not able to reach a definitive answer [75]. Based on expert opinion, the presence of widespread pain on PDs should at least alert clinicians to consider the possibility of performing a more specific psychological screening [45].

### Limitations

There are some methodological issues that should be considered in this study. First, several methods to compute the pain extent have been described, but a gold standard is not yet available. In this study, the Margolis rating scale was applied, which has previously been validated in patients with chronic pain [76] but never tested before in people with FS. Again, reliability of PDs was not specifically tested in the current study’s sample of patients with FS. Instead, PDs’ reliability was assumed to be good on the basis of a previous study in patients with temporomandibular disorders using the same recall period (one week) [45]. To date, no data exists on reliability or validity of PDs in people with FS, therefore further research is warranted. Furthermore, it is important to note that TS and CPM measurements were only performed on the affected shoulder but not on a remote location as recommended in current CPM testing guidelines [77]. Moreover, although participants needed to be stable in medication intake at least 4 weeks before the beginning of the study, medication intake was not considered as an exclusion criteria. This fact may have influenced the pain extent reported by the participants and therefore influenced the results of the current study. Lastly, there was a lack of control of potential confounder factors such as alcohol intake, smoking, physical activity and hand dominance, which may have affected the results of some pain sensitization measures (i.e., CPM and TS).

## 5. Conclusions

In conclusion, this study has shown that the most frequent site of symptoms in people with FS is the anterolateral region of the shoulder. In addition to local shoulder symptoms, some patients with FS reported more extended areas of pain. This was observed more often in women than men. Additionally, enlarged areas of pain were associated with higher levels of pain intensity, pain catastrophizing and pain sensitization measured with the CSI. Further evaluation of the reliability and validity of PDs in people with FS is needed before its use can be advocated in clinical practice.

## Figures and Tables

**Table 1:** Participants’ characteristics.

Characteristics of Patients with FS	Mean (SD) (*N* = 48)
Age (y)	54.5 (7.5)
Weight (kg)	66.8 (11.8)
Height (cm)	166.3 (7.5)
Pain duration (months)	7 (5.7) *
Extent of pain (%)	12 (8.8) *
**Direct measures of CS**	**Mean (SD) (*N* = 48)**
PPT affected shoulder	3.1 (2)
Temporal summation (TS)	73.1 (121.8)
Conditioned pain modulation (CPM)	−0.26 (0.83)
**Indirect measures of CS**	
Central Sensitization Inventory (CSI)	31.6 (16)
**Clinical symptoms**	
Current pain (VAS, 0–100)	22.8 (26.2)
Pain last 24 h	49.9 (26.8)
Shoulder Pain and Disability Index (SPADI)	58.5 (18.6)
Pain Catastrophizing Scale (PCS)	15.1 (9)
Pain Vigilance and Awareness Questionnaire (PVAQ)	22.8 (8.7)

FS, frozen shoulder; CS, central sensitization; PPT, pressure pain thresholds; VAS, Visual Analogue Scale; * median and interquartile range.

**Table 2:** Spearman correlation coefficients between the extent of pain (total area of pain extracted from frontal and dorsal body views) computed using pain drawings and measures of central sensitization and clinical symptoms in patients with frozen shoulder (*N* = 48).

Measures	Correlation with Pain Extent (r_s_)
Pain duration (y)	0.195
**Direct measures of CS**	**Mean (SD) (*N* = 48)**
PPT affected shoulder	−0.118
Temporal summation (TS)	−0.149
Conditioned pain modulation (CPM)	−0.138
**Indirect measures of CS**	
Central Sensitization Inventory (CSI)	0.358 *
**Clinical symptoms**	
Current pain (VAS, 0–100)	0.421 *
Pain last 24 h (VAS, 0–100)	0.057
Shoulder Pain and Disability Index (SPADI)	0.182
Pain Catastrophizing Scale (PCS)	0.307 *
Pain Vigilance and Awareness Questionnaire (PVAQ)	−0.252

FS, frozen shoulder; CS, central sensitization; PPT, pressure pain thresholds; VAS, Visual Analogue Scale; * correlation is significant at the 0.05 level.

## Data Availability

Data available on request due to privacy and ethical restrictions.

## References

[1] Neviaser T.J. (1987). Adhesive Capsulitis. Orthop. Clin. N. Am..

[2] Fields B.K., Skalski M.R., Patel D.B., White E.A., Tomasian A., Gross J.S., Matcuk G.R. (2019). Adhesive Capsulitis: Review of Imaging Findings, Pathophysiology, Clinical Presentation, and Treatment Options. Skeletal Radiol..

[3] Lundberg B.J. (1969). The Frozen Shoulder: Clinical and Radiographical Observations the Effect of Manipulation under General Anesthesia Structure and Glycosaminoglycan Content of the Joint Capsule Local Bone Metabolism. Acta Orthop. Scand..

[4] Zuckerman J.D., Rokito A. (2011). Frozen Shoulder: A Consensus Definition. J. Shoulder Elbow Surg..

[5] Jones S., Hanchard N., Hamilton S., Rangan A. (2013). A Qualitative Study of Patients’ Perceptions and Priorities When Living with Primary Frozen Shoulder. BMJ Open.

[6] International Association for the Study of Pain, IASP. https://www.iasp-pain.org/resources/terminology/.

[7] Struyf F., Meeus M. (2014). Current Evidence on Physical Therapy in Patients with Adhesive Capsulitis: What Are We Missing?. Clin. Rheumatol..

[8] Roy J.-S., Bouyer L.J., Langevin P., Mercier C. (2017). Beyond the Joint: The Role of Central Nervous System Reorganizations in Chronic Musculoskeletal Disorders. J. Orthop. Sports Phys. Ther..

[9] Sawyer E.E., McDevitt A.W., Louw A., Puentedura E.J., Mintken P.E. (2018). Use of Pain Neuroscience Education, Tactile Discrimination, and Graded Motor Imagery in an Individual with Frozen Shoulder. J. Orthop. Sports Phys. Ther..

[10] Mena-del Horno S., Balasch-Bernat M., Dueñas L., Reis F., Louw A., Lluch E. (2020). Laterality Judgement and Tactile Acuity in Patients with Frozen Shoulder: A Cross-Sectional Study. Musculoskelet. Sci. Pract..

[11] Louw A., Puentedura E.J., Reese D., Parker P., Miller T., Mintken P.E. (2017). Immediate Effects of Mirror Therapy in Patients with Shoulder Pain and Decreased Range of Motion. Arch. Phys. Med. Rehabil..

[12] Lluch-Girbés E., Dueñas L., Mena-del Horno S., Luque-Suarez A., Navarro-Ledesma S., Louw A. (2019). A Central Nervous System-Focused Treatment Approach for People with Frozen Shoulder: Protocol for a Randomized Clinical Trial. Trials.

[13] Breckenridge J.D., McAuley J.H., Ginn K.A. (2020). Motor Imagery Performance and Tactile Spatial Acuity: Are They Altered in People with Frozen Shoulder?. Int. J. Environ. Res. Public. Health.

[14] Kraal T., Lübbers J., van den Bekerom M.P.J., Alessie J., van Kooyk Y., Eygendaal D., Koorevaar R.C.T. (2020). The Puzzling Pathophysiology of Frozen Shoulders–a Scoping Review. J. Exp. Orthop..

[15] Rodeo S.A., Hannafin J.A., Tom J., Warren R.F., Wickiewicz T.L. (1997). Immunolocalization of Cytokines and Their Receptors in Adhesive Capsulitis of the Shoulder. J. Orthop. Res..

[16] Rohleder N. (2014). Stimulation of Systemic Low-Grade Inflammation by Psychosocial Stress. Psychosom. Med..

[17] Gao Y.-J., Ji R.-R. (2008). Activation of JNK Pathway in Persistent Pain. Neurosci. Lett..

[18] Nijs J., Loggia M.L., Polli A., Moens M., Huysmans E., Goudman L., Meeus M., Vanderweeën L., Ickmans K., Clauw D. (2017). Sleep Disturbances and Severe Stress as Glial Activators: Key Targets for Treating Central Sensitization in Chronic Pain Patients?. Expert Opin. Ther. Targets.

[19] Shraim M.A., Massé-Alarie H., Hodges P.W. (2021). Methods to Discriminate between Mechanism-Based Categories of Pain Experienced in the Musculoskeletal System: A Systematic Review. Pain.

[20] Nijs J., Torres-Cueco R., Van Wilgen P., Lluch Girbés E., Struyf F., Roussel N., Van Oosterwijck J., Daenen L., Kuppens K., Vanderweeen L. (2014). Applying Modern Pain Neuroscience in Clinical Practice: Criteria for the Classification of Central Sensitization Pain. Pain Physician.

[21] Graven-Nielsen T., Arendt-Nielsen L. (2010). Assessment of Mechanisms in Localized and Widespread Musculoskeletal Pain. Nat. Rev. Rheumatol..

[22] Arendt-Nielsen L., Skou S.T., Nielsen T.A., Petersen K.K. (2015). Altered Central Sensitization and Pain Modulation in the CNS in Chronic Joint Pain. Curr. Osteoporos. Rep..

[23] Middlebrook N., Rushton A.B., Abichandani D., Kuithan P., Heneghan N.R., Falla D. (2021). Measures of Central Sensitization and Their Measurement Properties in Musculoskeletal Trauma: A Systematic Review. Eur. J. Pain.

[24] Barbero M., Navarro-Santana M.J., Palacios-Ceña M., Ortega-Santiago R., Cescon C., Falla D., Fernández-de-Las-Peñas C. (2020). Clinical Significance and Diagnostic Value of Pain Extent Extracted from Pain Drawings: A Scoping Review. Diagnostics.

[25] Bertilson B., Grunnesjö M., Johansson S.-E., Strender L.-E. (2007). Pain Drawing in the Assessment of Neurogenic Pain and Dysfunction in the Neck/Shoulder Region: Inter-Examiner Reliability and Concordance with Clinical Examination. Pain Med..

[26] Falla D., Peolsson A., Peterson G., Ludvigsson M.L., Soldini E., Schneebeli A., Barbero M. (2016). Perceived Pain Extent Is Associated with Disability, Depression and Self-Efficacy in Individuals with Whiplash-Associated Disorders. Eur. J. Pain Lond. Engl..

[27] Uthaikhup S., Barbero M., Falla D., Sremakaew M., Tanrprawate S., Nudsasarn A. (2020). Profiling the Extent and Location of Pain in Migraine and Cervicogenic Headache: A Cross-Sectional Single-Site Observational Study. Pain Med..

[28] Hüllemann P., Keller T., Kabelitz M., Freynhagen R., Tölle T., Baron R. (2017). Pain Drawings Improve Subgrouping of Low Back Pain Patients. Pain Pract..

[29] Barbero M., Fernández-de-Las-Peñas C., Palacios-Ceña M., Cescon C., Falla D. (2017). Pain Extent Is Associated with Pain Intensity but Not with Widespread Pressure or Thermal Pain Sensitivity in Women with Fibromyalgia Syndrome. Clin. Rheumatol..

[30] Lluch Girbés E., Dueñas L., Barbero M., Falla D., Baert I.A.C., Meeus M., Sánchez-Frutos J., Aguilella L., Nijs J. (2016). Expanded Distribution of Pain as a Sign of Central Sensitization in Individuals With Symptomatic Knee Osteoarthritis. Phys. Ther..

[31] Willett M.J., Siebertz M., Petzke F., Erlenwein J., Rushton A., Soldini E., Barbero M., Falla D. (2020). The Extent of Pain Is Associated With Signs of Central Sensitization in Patients With Hip Osteoarthritis. Pain Pract. Off. J. World Inst. Pain.

[32] Gatchel R.J., Peng Y.B., Peters M.L., Fuchs P.N., Turk D.C. (2007). The Biopsychosocial Approach to Chronic Pain: Scientific Advances and Future Directions. Psychol. Bull..

[33] Laisné F., Lecomte C., Corbière M. (2012). Biopsychosocial Predictors of Prognosis in Musculoskeletal Disorders: A Systematic Review of the Literature (Corrected and Republished). Disabil. Rehabil..

[34] Leeuw M., Goossens M.E.J.B., Linton S.J., Crombez G., Boersma K., Vlaeyen J.W.S. (2007). The Fear-Avoidance Model of Musculoskeletal Pain: Current State of Scientific Evidence. J. Behav. Med..

[35] Sullivan M.J.L., Stanish W., Waite H., Sullivan M., Tripp D.A. (1998). Catastrophizing, Pain, and Disability in Patients with Soft-Tissue Injuries. Pain.

[36] McWilliams L.A., Cox B.J., Enns M.W. (2003). Mood and Anxiety Disorders Associated with Chronic Pain: An Examination in a Nationally Representative Sample. Pain.

[37] Chiaramonte R., Bonfiglio M., Chisari S. (2020). A Significant Relationship between Personality Traits and Adhesive Capsulitis. Rev. Assoc. Medica Bras..

[38] von Elm E., Altman D.G., Egger M., Pocock S.J., Gøtzsche P.C., Vandenbroucke J.P., STROBE Initiative (2014). The Strengthening the Reporting of Observational Studies in Epidemiology (STROBE) Statement: Guidelines for Reporting Observational Studies. Int. J. Surg. Lond. Engl..

[39] Kelley M.J., Mcclure P.W., Leggin B.G. (2009). Frozen Shoulder: Evidence and a Proposed Model Guiding Rehabilitation. J. Orthop. Sports Phys. Ther..

[40] Kuppens K., Hans G., Roussel N., Struyf F., Fransen E., Cras P., Van Wilgen C.P., Nijs J. (2018). Sensory Processing and Central Pain Modulation in Patients with Chronic Shoulder Pain: A Case-Control Study. Scand. J. Med. Sci. Sports.

[41] Cuesta-Vargas A.I., Roldan-Jimenez C., Neblett R., Gatchel R.J. (2016). Cross-Cultural Adaptation and Validity of the Spanish Central Sensitization Inventory. SpringerPlus.

[42] García Campayo J., Rodero B., Alda M., Sobradiel N., Montero J., Moreno S. (2008). Validation of the Spanish version of the Pain Catastrophizing Scale in fibromyalgia. Med. Clin..

[43] Esteve R., Ramírez-Maestre C., López-Martínez A.E. (2013). Empirical Evidence of the Validity of the Spanish Version of the Pain Vigilance Awareness Questionnaire. Int. J. Behav. Med..

[44] Beales D., Mitchell T., Moloney N., Rabey M., Ng W., Rebbeck T. (2021). Masterclass: A Pragmatic Approach to Pain Sensitivity in People with Musculoskeletal Disorders and Implications for Clinical Management for Musculoskeletal Clinicians. Musculoskelet. Sci. Pract..

[45] Barbero M., Moresi F., Leoni D., Gatti R., Egloff M., Falla D. (2015). Test-Retest Reliability of Pain Extent and Pain Location Using a Novel Method for Pain Drawing Analysis. Eur. J. Pain.

[46] Margolis R.B., Tait R.C., Krause S.J. (1986). A Rating System for Use with Patient Pain Drawings. Pain.

[47] Rolke R., Baron R., Maier C., Tölle T.R., Treede D.R., Beyer A., Binder A., Birbaumer N., Birklein F., Bötefür I.C. (2006). Quantitative Sensory Testing in the German Research Network on Neuropathic Pain (DFNS): Standardized Protocol and Reference Values. Pain.

[48] Rolke R., Andrews Campbell K., Magerl W., Treede R.-D. (2005). Deep Pain Thresholds in the Distal Limbs of Healthy Human Subjects. Eur. J. Pain.

[49] Doménech-García V., Palsson T.S., Herrero P., Graven-Nielsen T. (2016). Pressure-Induced Referred Pain Is Expanded by Persistent Soreness. Pain.

[50] Yarnitsky D., Arendt-Nielsen L., Bouhassira D., Edwards R.R., Fillingim R.B., Granot M., Hansson P., Lautenbacher S., Marchand S., Wilder-Smith O. (2010). Recommendations on Terminology and Practice of Psychophysical DNIC Testing. Eur. J. Pain.

[51] Staud R., Robinson M.E., Price D.D. (2007). Temporal Summation of Second Pain and Its Maintenance Are Useful for Characterizing Widespread Central Sensitization of Fibromyalgia Patients. J. Pain.

[52] Cathcart S., Winefield A.H., Rolan P., Lushington K. (2009). Reliability of Temporal Summation and Diffuse Noxious Inhibitory Control. Pain Res. Manag..

[53] Kennedy D.L., Kemp H.I., Ridout D., Yarnitsky D., Rice A.S.C. (2016). Reliability of Conditioned Pain Modulation: A Systematic Review. Pain.

[54] Scerbo T., Colasurdo J., Dunn S., Unger J., Nijs J., Cook C. (2018). Measurement Properties of the Central Sensitization Inventory: A Systematic Review. Pain Pract. Off. J. World Inst. Pain.

[55] Neblett R., Hartzell M.M., Mayer T.G., Cohen H., Gatchel R.J. (2017). Establishing Clinically Relevant Severity Levels for the Central Sensitization Inventory. Pain Pract. Off. J. World Inst. Pain.

[56] Boonstra A.M., Schiphorst Preuper H.R., Reneman M.F., Posthumus J.B., Stewart R.E. (2008). Reliability and Validity of the Visual Analogue Scale for Disability in Patients with Chronic Musculoskeletal Pain. Int. J. Rehabil. Res. Int. Z. Rehabil. Rev. Int. Rech. Readapt..

[57] Lee J.S., Hobden E., Stiell I.G., Wells G.A. (2003). Clinically Important Change in the Visual Analog Scale after Adequate Pain Control. Acad. Emerg. Med. Off. J. Soc. Acad. Emerg. Med..

[58] Membrilla-Mesa M.D., Cuesta-Vargas A.I., Pozuelo-Calvo R., Tejero-Fernández V., Martín-Martín L., Arroyo-Morales M. (2015). Shoulder Pain and Disability Index: Cross Cultural Validation and Evaluation of Psychometric Properties of the Spanish Version. Health Qual. Life Outcomes.

[59] Roach K.E., Budiman-Mak E., Songsiridej N., Lertratanakul Y. (1991). Development of a Shoulder Pain and Disability Index. Arthritis Care Res. Off. J. Arthritis Health Prof. Assoc..

[60] Roy J.-S., MacDermid J.C., Woodhouse L.J. (2009). Measuring Shoulder Function: A Systematic Review of Four Questionnaires. Arthritis Rheum..

[61] Sullivan M., Bishop S., Pivik J. (1995). The Pain Catastrophizing Scale: Development and Validation. Psychol. Assess..

[62] Pilar Martínez M., Miró E., Sánchez A.I., Lami M.J., Prados G., Ávila D. (2015). Spanish Version of the Pain Vigilance and Awareness Questionnaire: Psychometric Properties in a Sample of Women with Fibromyalgia. Span. J. Psychol..

[63] Munro B.H. (2005). Statistical Methods for Health Care Research.

[64] Gumina S., Candela V., Passaretti D., Venditto T., Carbone S., Arceri V., Giannicola G. (2014). Intensity and Distribution of Shoulder Pain in Patients with Different Sized Postero-Superior Rotator Cuff Tears. J. Shoulder Elbow Surg..

[65] Bayam L., Ahmad M.A., Naqui S.Z., Chouhan A., Funk L. (2011). Pain Mapping for Common Shoulder Disorders. Am. J. Orthop..

[66] Robinson M.E., Gagnon C.M., Riley J.L., Price D.D. (2003). Altering Gender Role Expectations: Effects on Pain Tolerance, Pain Threshold, and Pain Ratings. J. Pain.

[67] Thorn B.E., Clements K.L., Ward L.C., Dixon K.E., Kersh B.C., Boothby J.L., Chaplin W.F. (2004). Personality Factors in the Explanation of Sex Differences in Pain Catastrophizing and Response to Experimental Pain. Clin. J. Pain.

[68] Wood L.R.J., Peat G., Thomas E., Duncan R. (2007). Knee Osteoarthritis in Community-Dwelling Older Adults: Are There Characteristic Patterns of Pain Location?. Osteoarthr. Cartil..

[69] Ris I., Barbero M., Falla D., Larsen M.H., Kraft M.N., Søgaard K., Juul-Kristensen B. (2019). Pain Extent Is More Strongly Associated with Disability, Psychological Factors, and Neck Muscle Function in People with Non-Traumatic versus Traumatic Chronic Neck Pain: A Cross Sectional Study. Eur. J. Phys. Rehabil. Med..

[70] Gerhardt A., Eich W., Treede R.-D., Tesarz J. (2017). Conditioned Pain Modulation in Patients with Nonspecific Chronic Back Pain with Chronic Local Pain, Chronic Widespread Pain, and Fibromyalgia. Pain.

[71] Gruener H., Zeilig G., Laufer Y., Blumen N., Defrin R. (2016). Differential Pain Modulation Properties in Central Neuropathic Pain after Spinal Cord Injury. Pain.

[72] Pande K.C., Tripathi S., Kanoi R. (2005). Limited Clinical Utility of Pain Drawing in Assessing Patients with Low Back Pain. J. Spinal Disord. Tech..

[73] Carnes D., Ashby D., Underwood M. (2006). A Systematic Review of Pain Drawing Literature: Should Pain Drawings Be Used for Psychologic Screening?. Clin. J. Pain.

[74] Asmundson G.J.G., Katz J. (2009). Understanding the Co-Occurrence of Anxiety Disorders and Chronic Pain: State-of-the-Art. Depress. Anxiety.

[75] Reis F., Guimarães F., Nogueira L.C., Meziat-Filho N., Sanchez T.A., Wideman T. (2019). Association between Pain Drawing and Psychological Factors in Musculoskeletal Chronic Pain: A Systematic Review. Physiother. Theory Pract..

[76] Margolis R.B., Chibnall J.T., Tait R.C. (1988). Test-Retest Reliability of the Pain Drawing Instrument. Pain.

[77] Yarnitsky D., Bouhassira D., Drewes A.M., Fillingim R.B., Granot M., Hansson P., Landau R., Marchand S., Matre D., Nilsen K.B. (2015). Recommendations on Practice of Conditioned Pain Modulation (CPM) Testing. Eur. J. Pain.

